# MiR-204-3p Inhibited the Proliferation of Bladder Cancer Cells via Modulating Lactate Dehydrogenase-Mediated Glycolysis

**DOI:** 10.3389/fonc.2019.01242

**Published:** 2019-11-29

**Authors:** Jinan Guo, Pan Zhao, Zengqin Liu, Zaishang Li, Yeqing Yuan, Xueqi Zhang, Zhou Yu, Jiequn Fang, Kefeng Xiao

**Affiliations:** ^1^Department of Urology, Shenzhen Urology Minimally Invasive Engineering Center, The Second Clinical Medical College of Jinan University, Shenzhen People's Hospital, The First Affiliated Hospital of South University of Science and Technology of China, Shenzhen, China; ^2^Shenzhen Public Service Platform on Tumor Precision Medicine and Molecular Diagnosis, Shenzhen Urology Minimally Invasive Engineering Center, The Second Clinical Medical College of Jinan University, Shenzhen People's Hospital, The First Affiliated Hospital of South University of Science and Technology of China, Shenzhen, China; ^3^Clinical Medical Research Center, Shenzhen Urology Minimally Invasive Engineering Center, The Second Clinical Medical School of Jinan University, Shenzhen People's Hospital, The First Affiliated Hospital of South University of Science and Technology of China, Shenzhen, China

**Keywords:** bladder cancer, miR-204-3p, lactate dehydrogenase, glycolysis, proliferation

## Abstract

MicroRNAs (miRNAs) are endogenous non-coding RNAs that negatively regulate the expression of downstream targeted mRNAs. Increasing evidence has suggested that miRNAs act as tumor suppressors or oncogenes to interfere the progression of cancers. Here, we showed that miR-204-3p was decreased in bladder cancer tissues and cell lines. Down-regulation of miR-204-3p was significantly associated with a poor prognosis in bladder cancer patients. Overexpression of miR-204-3p inhibited proliferation and induced apoptosis in bladder cancer cells. Furthermore, miR-204-3p was found to bind to the 3′-untranslated region (UTR) of the lactate dehydrogenase (LDHA), which consequently reduced the expression of both mRNA and protein of LDHA. Interestingly, overexpression of miR-204-3p decreased glucose consumption and lactate production of bladder cancer cells. Overexpression of LDHA relieved the growth inhibition and cell apoptosis enhancement by miR-204-3p in bladder cancer cells. These results demonstrated that miR-204-3p negatively modulated the proliferation of bladder cancer cells via targeting LDHA-mediated glycolysis. MiR-204-3p might be a promising candidate for designing anticancer medication.

## Introduction

Bladder cancer (BC) is one of the most common malignancies in the urological system (1). There are approximately 356,000 new cases and 145,000 deaths annually around the world (1–3). The high occurrence and mortality of BC make it a big threat to the health of patients. Although remarkable progress has been made in the treatment of BC including surgical resection and chemotherapy, the 5-year overall survival of BC remains poor (4, 5). Therefore, it is urgent to identify novel biomarkers and targets of BC.

MicroRNAs (miRNAs) are a class of single-stranded non-coding RNAs with the length of 22–24 nucleotides (6, 7). MiRNAs negatively regulate the gene expression via binding with the 3′-untranslated region (UTR) of targets, which leads to the degradation or translation inhibition of mRNAs (8). MiRNAs are reported to be involved in both physiological and pathological conditions including cell proliferation, differentiation, and apoptosis (9, 10). Recent research found that aberrant expression of miRNAs was associated with the tumorigenesis of BCs (9–12). Therefore, detecting the abundance of miRNA might provide novel evidence for the diagnosis of BC and benefit the outcome of cancer patients. To get a whole picture about the expression of miRNAs in BC, we reviewed the previous publications relevant to the altered miRNAs in BC and performed a meta-analysis. The data showed that miR-204-3p was significantly down-regulated in the urine supernatant of BC patients (13). Interestingly, miR-204-3p was frequently found to be down-regulated in cancers, which acted as a tumor suppressor in tumorigenesis (14). Abnormal expression of miR-204-3p was observed in gastrointestinal stromal tumors, breast cancer, and hepatocellular carcinoma (HCC) (15, 16). However, the expression and function of miR-204-3p in BC remain largely unknown.

Aerobic glycolysis has been considered as a distinct hallmark of cancer cells, which represents that cancer cells addictively depend on glycolysis to metabolize glucose even in oxygen-rich condition (17–19). Therefore, understanding the mechanisms that contribute to the glycolysis process will provide novel cues for therapeutic strategies for cancer treatment. The lactate dehydrogenase (LDHA) is a critical enzyme of the glycolysis, which catalyzes the production of lactate (20). Up-regulation of LDHA has been found in a variety of human cancers, which is associated with cell growth, metastasis, and poor prognosis of cancer patients (21–24). Notably, miRNAs are reported to target LDHA and negatively regulate the expression of LDHA. For example, repression of LDHA by miR-34a inhibited the glycolysis and suppressed the growth of breast cancer cells (20). Recent study showed that miR-142-3p targeted LDHA, which reduced the glycolysis and growth of HCC cells (25). These reports suggest that the negative modulation of LDHA by miRNA is an important strategy to regulate the progression of cancers.

In this study, we investigated the function of miR-204-3p in BC. The data showed that miR-204-3p was down-regulated in BC tissues and cell lines. Overexpression of miR-204-3p decreased the proliferation of BC cells via modulating LDHA-mediated glycolysis.

## Materials and Methods

### Meta-Analysis

The studies that reported the alternation of miRNAs in BCs was searched using the National Center for Biotechnology Information (NCBI) Gene Expression Omnibus (GEO) and EBI ArrayExpress databases with the keywords “human” and “bladder” and “miRNA.” For a meta-analysis, priority was given to the peer-reviewed publications that investigated the expression of miRNAs in both the BC and corresponding normal controls. For the studies without data of control group, only cell-culture-based results were excluded. Data extraction from the selected publications contained the first author's name, publication time, sample size, patient's age, type of case, origin of the studied population, tumor stage, detection method, and cutoff values for down-regulation or up-regulation. The quality of the publications was evaluated by the Newcastle-Ottawa scale, which was generally used for assessing the quality of non-randomized studies in meta-analyses. Each article was subjected to assessment with eight methodology items with the score ranging from 0 to 9. The higher score indicated better quality of the publication. The articles with score of 7 or more were recruited for the meta-analysis.

### Clinical Samples and Cell Lines

A total of 60 paired BC and adjacent normal tissues were obtained from the BC patients who underwent surgery in the Shenzhen People's Hospital. The tissues were confirmed by three pathologists independently. None of the patients received radiotherapy or chemotherapy before the surgical resection. All the tissues were maintained in liquid nitrogen until use. All the experimental procedures were approved by the Research Ethics Committee of Shenzhen People's Hospital, and each patient signed the written informed consent.

The normal human urothelial SV-HUC-1 cells were purchased from the Institute of Cell Research, Chinese Academy of Sciences (Shanghai, China). BC cell lines including SW780, J82, UMUC3, 5637, and T24 were purchased from the American Type Culture Collection (ATCC, Rockville, MD, USA). Cells were cultured in Roswell Park Memorial Institute (RPMI)-1640 medium (Gibco, Thermo Fisher Scientific, Carlsbad, CA, USA) supplemented with 10% fetal bovine serum (FBS; Sigma-Aldrich, St. Louis, MO, USA), 100 U/ml of penicillin and 100 mg/ml of streptomycin (Invitrogen, Carlsbad, CA, USA) at 37°C with 5% CO_2_.

### Reverse Transcriptase Quantitative PCR Analysis

MiRNAs were extracted from the cell lines or bladder tissues using the miRNeasy Mini Kit (QIAGEN, Hilden, Germany) according to the manufacturer's instruction; 0.5 μg of RNA was reversely transcripted into cDNA with the PrimeScript RT Master Mix (Takara, Dalian, China). Real-time PCR analysis was performed with the SYBR Select Master Mix (Takara, Dalian, China) on the ABI7500 platform (Applied Biosystems, Foster City, CA, USA). The expression of U6 RNA and GAPDH was detected as the respective endogenous control. The expression level of miR-204-3p and LDHA was normalized to U6 and GAPDH and calculated using the 2^−ΔΔCT^ method. The primers were as follows: miR-204-3p, F, 5′-ACACTCCAGCTGGGGCTGGGAAGGCAAAGGG-3′ and R, 5′-CTCAACTGGTGTCGTGGA-3′; LDHA, F, 5′-AGCCCGATTCCGTTACCT-3′ and R, 5′-CACCAGCAACATTCATTCCA-3′; U6, F, 5′-CTCGCTTCGGCAGCACA-3′ and R, 5′-AACGCTTCACGAATTTGCGT-3′; and GAPDH, F, 5′-TGACGCTGGGGCTGGCATTG-3′ and R, 5′-GCTCTTGCTGGGGCTGGTGG-3′.

### Cell Viability

The proliferation of BC cells with the transfection of control miRNA or miR-204-3p mimics was evaluated by the Cell Counting Kit-8 assay (CCK-8, Beyotime, Shanghai, China). Briefly, 10 μl of CCK-8 reagent was added into the medium at the indicated time points of 1, 2, 3, 4, and 5 days and incubated for 4 h at 37°C. The absorbance value (optical density [OD]) of each well at 450 nm was detected with a microplate reader (Bio-Rad, CA, USA). The results were obtained from three independent experiments.

### Cell Colony Formation

The BC cells expressing the indicated miRNA were seeded in a 6-well plate with the density of 1,000 cells per well. Cells were cultured with the RPMI-1640 medium for 2 weeks. And then the medium was discarded, and the cells were washed twice with phosphate-buffered saline (PBS). Cells were fixed with methanol for 15 min at room temperature (RT). After being washed with PBS, the colonies were stained with 1% crystal violet, and the number of colonies was counted with light microscopy.

### Western Blot

Protein was extracted from the BC cells with the NP-40 lysis buffer (150 mM of NaCl, 1% NP-40, 50 mM of Tris–HCl (pH 8.0), and 1 mM of EDTA) in the presence of proteinase inhibitor (Millipore, Braunschweig, Germany). The protein concentration was evaluated using the bicinchoninic acid (BCA) assay kit (Beyotime, Shanghai, China); 15 μg of proteins was loaded and separated by the 15% sodium dodecyl sulfate–polyacrylamide gel electrophoresis (SDS-PAGE) and then transferred onto the polyvinylidene difluoride (PVDF) membrane (Millipore, Braunschweig, Germany). The membrane was firstly blocked with 5% non-fat milk at RT for 1 h, followed by incubation with primary antibodies at RT for 2 h and by incubation of the membrane with goat anti-mouse IgG secondary antibody (1:3,000, Bio-Rad, CA, USA), and then analyzed using the chemiluminescent horseradish peroxidase (HRP) conjugated substrate (Millipore, Braunschweig, Germany). GAPDH was used as the loading control. Antibodies including anti-LDHA (#2012, 1:3,000, Cell Signaling Technology, Inc., Danvers, MA, USA) and anti-GAPDH (#5174, 1:3,000, Cell Signaling Technology, Inc., Danvers, MA, USA) were commercially obtained.

### Luciferase Reporter Assay

The wild-type (WT) or mutant (MUT) 3′-UTR of LDHA containing the putative binding sites of miR-204-3p was inserted into the pmiR-GLO vector. BC cells were transfected with the control miRNA or miR-204-3p mimics in the presence of luciferase reporter vector. After transfection for 48 h, cells were harvested, and the luciferase activity was determined with the dual-luciferase reporter system (Promega, Madison, WI, USA). The experiment was performed in triplicate.

### Measurement of the Glucose Consumption and Lactate Production

BC cells transfected with the corresponding miRNAs were cultured in RPMI-1640 medium without phenol red (Gibco, New York, NY, USA) for 24 h, and then the medium was collected. The glucose consumption and lactate production were determined using the Glucose Assay Kit (GAHK20-1KT; Sigma-Aldrich, USA) and Lactate Assay Kit (BioVision, CA, USA) according to manufacturers' instructions, respectively. The protein level in each group was measured using the BCA assay kit (Beyotime, Shanghai, China) for the normalization.

### Statistical Analysis

Results were presented as mean ± standard deviation from three independent experiments. The data were analyzed with the SPSS 19.0 software (SPSS Inc., Chicago, IL, USA). Significant differences between/among treatment groups were analyzed using unpaired Student's *t*-test or one-way ANOVA followed by Dunnett's *post hoc* test. *P* < 0.05 was considered to be statistically significant.

## Results

### MiR-204-3p Was Down-Regulated in Bladder Cancer Tissues and Cell Lines

**Figure 1:**
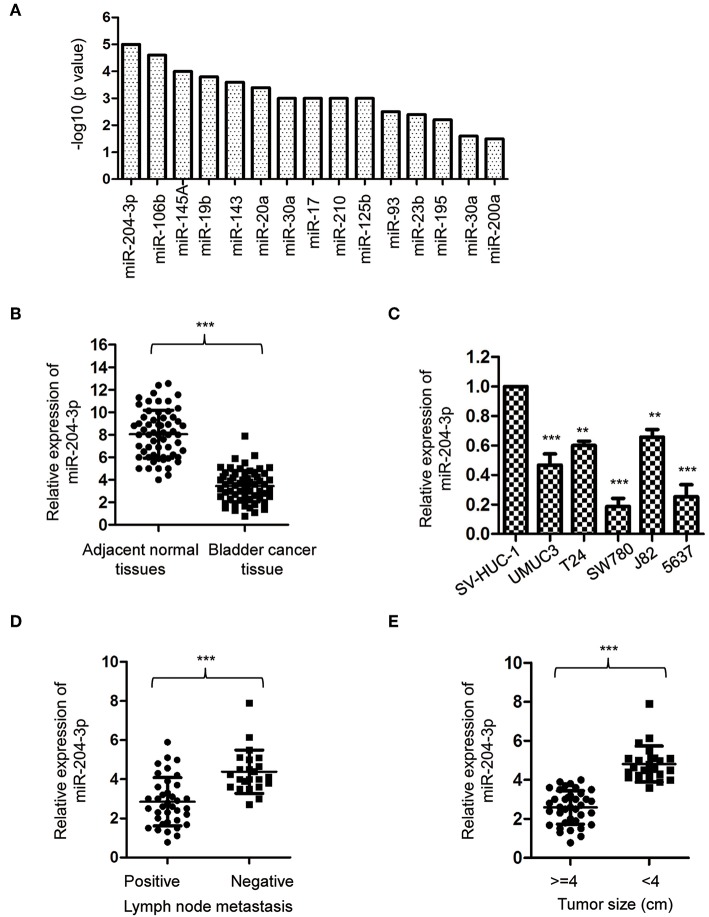
MiR-204-3p was decreased in bladder cancer. **(A)** Meta-analysis on the aberrantly expressed miRNAs in bladder cancer patients. **(B)** The expression of miR-204-3p in 60 paired bladder cancer tissues and the corresponding adjacent normal tissues was determined by RT-qPCR. **(C)** The expression of miR-204-3p in bladder cancer cells and normal bladder epithelial cell SV-HUC-1 was determined by the RT-qPCR analysis. **(D)** The expression of miR-204-3p in the bladder cancer patients with or without lymph node metastasis was determined by RT-qPCR. **(E)** The expression of miR-204-3p in bladder cancer tissues stratified by tumor size was determined by RT-qPCR. **P < 0.01 and ***P < 0.001. RT-qPCR, reverse transcriptase quantitative PCR.

To obtain a whole picture of miRNA expression in BC, a miRNA meta-analysis was performed using previous publications. The data found that miR-204-3p was significantly aberrantly expressed in the urine supernatant of the BC patients (Figure 1A). To confirm this result, the expression of miR-204-3p was detected by reverse transcriptase quantitative PCR (RT-qPCR) with paired BC tissues and corresponding normal tissues. The result showed that the level of miR-204-3p was significantly decreased in BC tissues in comparison with that of the normal counterparts (Figure 1B). To further confirm the aberrant expression of miR-204-3p in BC, the abundance of miR-204-3p in several BC cell lines was examined. As indicated in Figure 1C, miR-204-3p was remarkably decreased in BC cells, compared with the normal cells, including SW780, J82, UMUC3, 5637, and T24. These results suggested the down-regulation of miR-204-3p in BC. To further characterize the association between miR-204-3p expression and the clinicopathological features, the abundance of miR-204-3p in BC patients with or without lymph node metastasis was compared. The data revealed that lower expression of miR-204-3p was correlated with positive lymph node metastasis (Figure 1D). Consistently, decreased expression of miR-204-3p was also observed in BC patients with larger tumor size (Figure 1E). These results demonstrated that miR-204-3p was down-regulated in BC and associated with a poor prognosis of cancer patients.

### Overexpression of MiR-204-3p Inhibited the Proliferation and Induced Apoptosis of Bladder Cancer Cells

**Figure 2:**
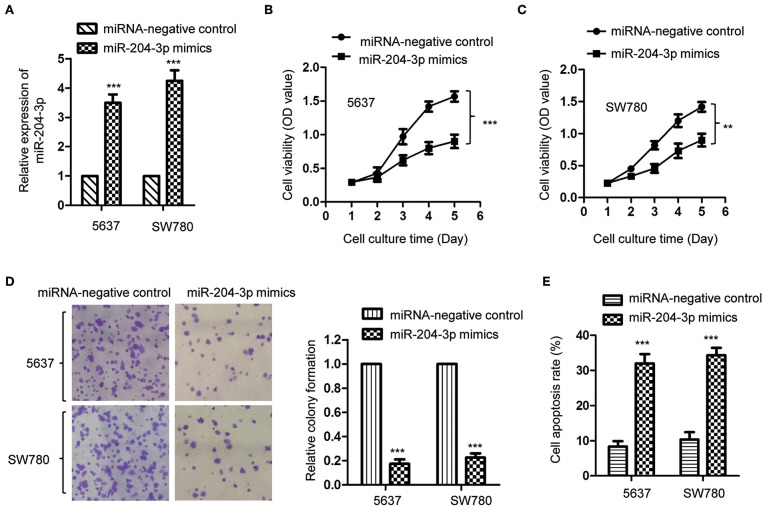
Overexpression of miR-204-3p inhibited the proliferation of bladder cancer cells. **(A)** The expression of miR-204-3p in both SW780 and 5637 cells with the transfection of miR-204-3p mimics or control miRNA was validated by RT-qPCR. **(B,C)** The proliferation of cells with miR-204-3p mimics or control miRNA transfection was determined by CCK-8 assay. **(D)** Colony formation assay was performed in both SW780 and 5637 cells with the transfection of miR-204-3p mimics or control miRNA. **(E)** Flow cytometry was performed in both SW780 and 5637 cells with the transfection of miR-204-3p or control miRNA. *N* = 3; **P < 0.01 and ***P < 0.001. RT-qPCR, reverse transcriptase quantitative PCR; CCK-8, Cell Counting Kit-8.

As miR-204-3p was decreased in BC, to investigate the effect of aberrant expression of miR-204-3p on the growth of BC cells, SW780 and 5637, which harbored the lowest expression of miR-204-3p among all the cell lines we tested (Figure 1C), were transfected with miR-204-3p mimics to up-regulate the expression of miR-204-3p. The overexpression of miR-204-3p in both SW780 and 5637 cells was detected by RT-qPCR (Figure 2A). The influence of miR-204-3p on the proliferation of BC cells was determined by CCK-8 assay. As showed in Figures 2B,C, the growth of both SW780 and 5637 cells was significantly inhibited with overexpressed miR-204-3p. Consistently, the colony formation assay indicated that overexpression of miR-204-3p dramatically decreased the number of colonies compared with those of the control cells (Figure 2D). In addition, to further confirm the negative regulation of overexpressed miR-204-3p on the growth of BC cells, the apoptosis rate of both SW780 and 5637 cells was examined with the fluorescence-activated cell sorting (FACS) analysis. The result suggested that ectopic expression of miR-204-3p significantly enhanced the apoptosis of BC cells (Figure 2E). These data demonstrated that overexpression of miR-204-3p inhibited the growth of BC cells, which suggested the potential tumor suppressive function of miR-204-3p in BC.

### Lactate Dehydrogenase Was a Target of MiR-204-3p in Bladder Cancer Cells

**Figure 3:**
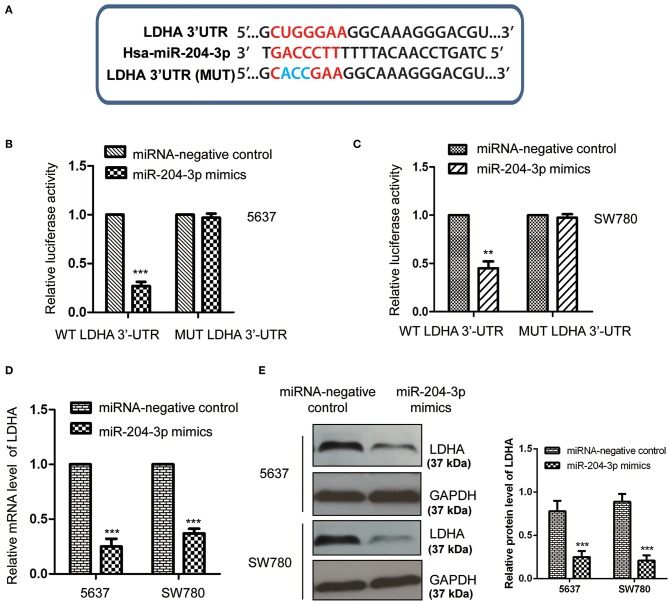
MiR-204-3p targeted LDHA in bladder cancer cells. **(A)** The complementary sequences in the 3′-UTR of LDHA and miR-204-3p. **(B,C)** The effects of miR-204-3p on the luciferase activity of wild-type or mutant LDHA 3′-UTR were determined by luciferase reporter assay. **(D,E)** The effects of miR-204-3p overexpression on the mRNA and protein levels of LDHA in both SW780 and 5637 cells were validated by RT-qPCR and western blot assay, respectively. *N* = 3; **P < 0.01 and ***P < 0.001. LDHA, lactate dehydrogenase; UTR, untranslated region; RT-qPCR, reverse transcriptase quantitative PCR.

To further explore the underlying molecular mechanism by which miR-204-3p modulated the growth of BC cells, the targets of miR-204-3p were predicted using the miRDB database (http://mirdb.org). Among the candidates, LDHA was found as a possible target of miR-204-3p (Figure 3A). To confirm the potential binding between miR-204-3p with the LDHA 3′-UTR (position 515-521), the WT or MUT 3′-UTR of LDHA containing the putative binding sites of miR-204-3p was inserted into the pmiR-GLO vector. Luciferase reporter assay was performed by co-transfecting negative control miRNA or miR-204-3p mimics with WT or MUT 3′-UTR of LDHA. The results showed that overexpression of miR-204-3p significantly reduced the luciferase activity of the WT 3′-UTR of LDHA; however, no remarkable decrease was observed when cells were transfected with MUT 3′-UTR of LDHA (Figures 3B,C). This result indicated that miR-204-3p bound the 3′-UTR of LDHA.

To detect whether the binding of miR-204-3p at the 3′-UTR affected the mRNA stability of LDHA, both SW780 and 5637 cells were transfected with control miRNA or miR-204-3p mimics, and the mRNA level of LDHA was examined with the RT-qPCR assay. As shown in Figure 3D, overexpression of miR-204-3p significantly reduced the mRNA level of LDHA in BC cells. Additionally, the protein level of LDHA in SW780 and 5637 cells expressing miR-204-3p was also investigated by Western blot using the anti-LDHA antibody. The data showed that the level of LDHA was down-regulated with the transfection of miR-204-3p in SW780 and 5637 cells (Figure 3E). These results demonstrated that LDHA was a target of miR-204-3p and negatively regulated by miR-204-3p in BC cells.

### MiR-204-3p Suppressed the Glycolysis of Bladder Cancer Cells by Modulating Lactate Dehydrogenase

**Figure 4:**
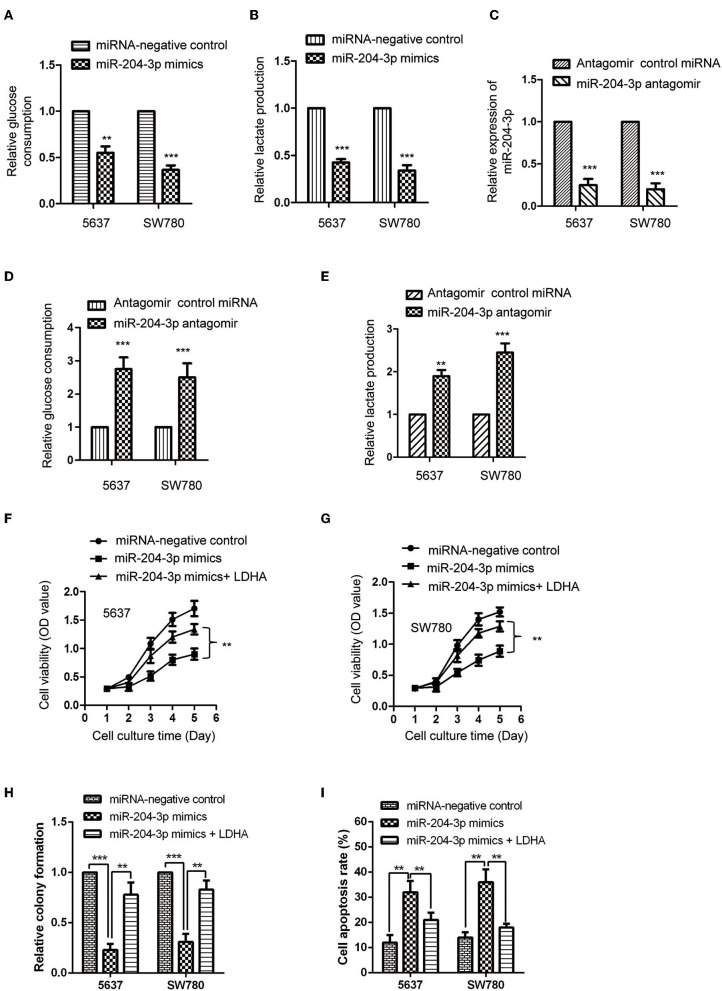
MiR-204-3p modulated the glycolysis of bladder cancer cells. **(A,B)** SW780 and 5637 cells were transfected with control miRNA or miR-204-3p mimics, and the glucose consumption and lactate production were measured. **(C)** The expression of miR-204-3p in SW780 and 5637 cells with antagomir control miRNA or miR-204-3p antagomir transfection was determined by the RT-qPCR. **(D,E)** The effects of miR-204-3p knockdown on the glucose uptake and lactate generation ofthe SW780 and 5637 cells were determined by Glucose Assay Kit and Lactate Assay Kit, respectively. **(F,G)** The cell viability of SW780 and 5637 with co-transfection with miRNAs and plasmid vectors was determined by CCK-8 assay. **(H)** The cell growth of SW780 and 5637 with co-transfection with miRNAs and plasmid vectors was determined by colony formation assay. **(I)** The cell apoptosis of SW780 and 5637 cells with co-transfection with miRNA and plasmid vectors was evaluated by flow cytometry. *N* = 3; **P < 0.01 and ***P < 0.001. RT-qPCR, reverse transcriptase quantitative PCR; CCK-8, Cell Counting Kit-8.

LDHA was critical for the glucose metabolism of cancer cells. Considering the negative regulation of miR-204-3p on the expression of LDHA, the influence of miR-204-3p on the glycolysis of BC cells was determined by measuring the glucose consumption and lactate production. The results showed that overexpression of miR-204-3p significantly decreased the glucose uptake of SW780 and 5637 cells (Figure 4A). Consistently, the generation of lactate of BC cells was also dramatically reduced in the presence of high level of miR-204-3p (Figure 4B). To further confirm these results, miR-204-3p was knocked down in SW780 and 5637 cells by transfecting miR-204-3p antagomir. The reduction of miR-204-3p was verified by RT-qPCR assay (Figure 4C). The glycolysis of BC cells harboring depleted miR-204-3p was explored. As shown in Figure 4D, down-regulation of miR-204-3p remarkably elevated the glucose consumption of both SW780 and 5637 cells. Increased lactate production was also obtained with the decreased expression of miR-204-3p (Figure 4E). These results indicated that miR-204-3p was a negative regulator of the glycolysis of BC cells.

To further investigate whether the inhibitory effect of miR-204-3p on the proliferation of BC cells was achieved via LDHA, CCK-8 assay was performed by transfecting LDHA in miR-204-3p overexpressed cells. The results showed that highly expressed miR-204-3p suppressed the proliferation and growth and induced cell apoptosis of both SW780 and 5637 cells, while rescue the expression of LDHA attenuated the inhibitory effect of miR-204-3p on the proliferation growth of BC cells and the enhanced effect of miR-204-3p on the cell apoptosis of BC cells (Figures 4F–I). These data demonstrated that miR-204-3p decreased the expression of LDHA, inhibited the glycolysis, and suppressed the growth of BC cells.

## Discussion

Although remarkable progression has been made in the treatment of BC with the application of surgery and chemotherapy, the prognosis of BC is still poor. Moreover, most patients are diagnosed at the advanced stage and showed unsatisfactory response to treatments. Notably, increasing evidence has suggested the critical roles of miRNAs in the initiation and progression of cancers (9–11). Here, we showed that miR-204-3p was decreased in both BC tissues and cell lines, which was associated with the poor prognosis of BC patients. Further gain-of-function analysis demonstrated that overexpression of miR-204-3p suppressed the growth of BC cells, which suggested the potential suppressive function of miR-204-3p in modulating the progression of BC.

The involvement of miR-204-3p in cancer has been highlighted by the studies that aberrant expression of miR-204-3p can be used to distinguish the malignant progression in gastric cancer (16) and breast cancer (26). Significant decrease of miR-204-3p was observed in HCC, which suppressed the growth of HCC via targeting fibronectin 1 (15). Recent study by Chen et al. showed that xanthohumol, a prenylated chalcone potential for anticancer therapy, up-regulated the expression of miR-204-3p in glioma cells and induced the cell apoptosis through modulating the insulin-like growth factor (IGF)-binding protein (27). In clear cell renal cell carcinoma (ccRCC), miR-204-3p was found to be down-regulated by the ERβ-suppressed circular RNA ATP2B1, which increased the expression of fibronectin 1 and promoted the invasion of ccRCC cells (28). These findings suggested the potential tumor suppressive function of miR-204-3p in cancers. In the present study, our data revealed that miR-204-3p was decreased in BC tissues compared with the normal tissue. Down-regulation of miR-204-3p was correlated with lymph node metastasis and larger tumor size. Further investigation might be of interest to explore the relationship between the expression of miR-204-3p with the 5-year overall survival of BC patients. Consistent with the decreased level of miR-204-3p in BC, overexpression of miR-204-3p significantly suppressed the proliferation and induced apoptosis of BC cells. *In vivo* tumorigenesis assay might be necessary to further confirm the modulating of miR-204-3p on the growth of BC cells in the future study.

The function of miRNAs was achieved via regulating the expression of downstream targets. In this study, the possible targets of miR-204-3p were predicted with the bioinformatics, and LDHA was identified as one of the binding partners of miR-204-3p. Overexpression of miR-204-3p significantly decreased both the mRNA and protein levels of LDHA in BC cells. Consistently, overexpression of miR-204-3p reduced the glucose consumption and lactate production of BC cells. LDHA deficiency results in a decrease in lactate production accompanied by inhibition of cell migration and invasion. Therefore, LDHA has been the target of different miRNAs in cancer cells to regulate the tumorigenesis. Among them, miR-142-3p was reported to target LDHA and inhibited the aerobic glycolysis of HCC (25). In breast cancer, miR-30-5p inhibited the cell growth and metastasis through suppression of LDHA-mediated glucose metabolism (29). These results collectively demonstrated that reprograming the glycolysis via targeting LDHA has been a promising way to modulate the progression of cancers.

Our study still presented several limitations. Firstly, the relationship between miR-204-3p and the overall survival of the BC patients has not been determined in the study, which needs further examination. Secondly, the present study lacks the *in vivo* animal studies, and the effects of miR-203-3p on the *in vivo* BC growth should be considered in future studies. Thirdly, the targets of miR-204-3p were not limited to LDHA, and other potential targets may be explored in future studies.

In conclusions, our results demonstrated that miR-204-3p was down-regulated in BC and correlated with the progression of the BC patients. Overexpression of miR-204-3p decreased the growth of BC cells via down-regulating LDHA, which consequently suppressed the glucose metabolism of BC cells. These data indicated that targeting the miR-204-3p-LDHA pathway might interfere with the tumorigenesis of cancer cells.

## Data Availability Statement

All datasets for this study are included in the article/supplementary material.

## Ethics Statement

The studies involving human participants were reviewed and approved by the Institutional Review Board of Shenzhen people's Hospital. The patients/participants provided their written informed consent to participate in this study.

## Author Contributions

JG and KX designed the whole study. JG, PZ, ZLiu, and ZLi performed the experiments. YY, XZ, and ZY performed the statistical analysis. JF and KX drafted the manuscript.

### Conflict of Interest

The authors declare that the research was conducted in the absence of any commercial or financial relationships that could be construed as a potential conflict of interest.
